# The prognosis of heart failure patients: Does sodium level play a significant role?

**DOI:** 10.1371/journal.pone.0207242

**Published:** 2018-11-08

**Authors:** Tamrat Befekadu Abebe, Eyob Alemayehu Gebreyohannes, Yonas Getaye Tefera, Akshaya Srikanth Bhagavathula, Daniel Asfaw Erku, Sewunet Admasu Belachew, Begashaw Melaku Gebresillassie, Tadesse Melaku Abegaz

**Affiliations:** 1 Department of Clinical Pharmacy, School of Pharmacy, College of Medicine and Health Sciences, University of Gondar, Gondar, Ethiopia; 2 Department of Learning, Informatics, Management, and Ethics (LIME), Karolinska Institutet, Solna, Sweden; 3 Department of Internal Medicine, United Arab Emirates University, Al Ain, United Arab Emirates; 4 School of Pharmacy, University of Queensland, Brisbane, Australia; Osaka University Graduate School of Medicine, JAPAN

## Abstract

**Introduction:**

Heart failure (HF), a major cardiovascular disorder, remains a grievous clinical condition regardless of advances in medical care. Hyponatremia is classified as a serum sodium concentration of <135 mEq/L, and the prevalence, clinical impact and prognostic factor of hyponatremia in heart failure patients varies widely. The current study was conducted with the aim of assessing the prevalence of hyponatremia in patients hospitalized with a diagnosis of HF and comparing baseline clinical characteristic of HF patients based on their sodium status. Survival difference between patients with hyponatremia and normonatremia was also assessed and the clinical prognostic indicators of overall mortality in HF patients were evaluated.

**Method:**

A retrospective cohort study was conducted to assess medical records of heart failure patients who were admitted to Gondar University Referral Hospital. Patients were categorized based on their sodium level status at their first admission to the internal medicine department. Each patient was assigned to either of the following groups: hyponatremia if sodium < 135 mmol/L, or normonatremia if sodium ≥ 135 mmol/L.

**Result:**

Among 388 participants, the prevalence of hyponatremia in the study cohorts was 51.03%. Kaplan-Meier survival curves showed that there was a significant difference in survival status of HF patients among the two cohorts (Log—Rank test, P <0.0001). Hence, patients with normal sodium levels had a higher chance of survival over hyponatremic patients. Multivariate Cox regression has revealed a statistically significant association of mortality with the following variables: advanced age (AHR = 1.035 (1.012–1.058), P = 0.003), hyponatremia (AHR = 4.003 (1.778–9.009), P = 0.001), higher creatinine level (AHR = 1.929 (1.523–2.443), P = <0.0001) and, prescription of angiotensin-converting enzyme inhibitors (AHR = 0.410 (0.199–0.842), P = 0.015) and spironolactone (AHR = 0.511 (0.275–0.949), P = 0.033.

**Conclusion:**

In conclusion, hyponatremia is one of the crucial factors in the clinical prognosis of heart failure patients. However, as other prognostic factors (i.e. medication, creatine level, and age) also played vital roles in overall survival, well-controlled clinical trials (complete with medication dosing, laboratory outputs and long-term prospective follow up) are required to further study the impact of hyponatremia in HF patient’s prognosis in low income nations.

## Introduction

Heart failure (HF), a major cardiovascular disorder, remains a grievous clinical condition regardless of advancement in medical care, representing a considerable health care burden and overall health expenditure [1]. Hyponatremia or low serum sodium level is typically defined as a serum sodium concentration of <135 mEq/L and is one of the most common biochemical disorders featured in heart failure patients, with a prevalence close to 25% [2–4]. HF affects cardiac output by either decreasing heart rate or reducing the stroke volume. The reduction of cardiac output subsequently causes arterial under–filling, which triggers the renin angiotensin aldosterone system (RAAS). Angiotensin II, the final product of the RAAS, activates aldosterone from the adrenal cortex, which results in the reabsorption of water and salt into the blood. The expansion of extracellular fluid ultimately results in hyponatremia[5–7].

Several observational studies and clinical trials have been conducted to assess the prognostic impact of serum sodium levels at -admission and during hospitalization of HF patients. Accordingly, a strong association is established between the mortality of HF patients and low serum sodium status at admission [8–10]. This association was attributed to cardiorenal insufficiency and the related decrease in water elimination but there is no current evidence on the prognostic impact of changes in serum sodium concentrations on patients’ overall prognosis. For instance, the EVEREST study on vasopressin antagonist, tolvaptan, which corrects hyponatremia in patients with HF, showed an improvement in serum sodium concentration and alleviated some signs and symptoms of heart failure, but no improvement has been observed in overall clinical outcomes [11]. Several observational studies reported similar findings, suggesting hyponatremia as a marker of a more severe clinical condition, but not a target for treatment or intervention [12, 13]. The clinical significance of hyponatremia acquired during hospitalization was highlighted by Goldsmith, as a predictive factor for increased mortality and readmission in patients with HF[14].

In the Ethiopian context, there has been an epidemiological transition in the burden of diseases from communicable to non-communicable diseases (NCD) in the past decade [15]. Cardiovascular diseases represents as a major NCD which impacts patients quality of life, disease comorbidity and mortality [15]. A recent global burden of disease study has shown that a crude death rate of 120.5 deaths per 100,000 people was attributed to cardiovascular diseases [15]. Furthermore, the age-standardized death rate from cardiovascular diseases was estimated to be around 350.0 deaths per 100,000 people [15]. Although these figures show staggering progress in the management of cardiovascular disorders, the Ethiopian ministry of health recognized the importance of managing these disorders and has promulgated policies in place to reduce the disease burden [15]. However, insufficient surveillance of cardiovascular diseases causes a major obstacle in assessing patients’ progress and the impact of the policy shift. In this regard, heart failure is one of the major clinical conditions that has been significantly overlooked and not studied comprehensively.

The current study was, therefore, conducted with the aim of assessing the prevalence of hyponatremia in patients hospitalized with a diagnosis of HF and comparing baseline clinical characteristic of HF patients based on their sodium level status. Further, survival progress between patients with hyponatremia and normonatremia was assessed and clinical prognostic markers of overall mortality in HF patients were evaluated.

## Method

The current study was based on data collected for two previous studies authored by Abebe et al. [16, 17]. However, the study durations, study sample sizes and the main objectives differed in each study as the previous studies focused on the impact of ejection fraction on the prognosis of heart failure patients and the influence of anemia on the survival of heart failure patients.

Medical records of patients admitted to Gondar University Referral Hospital (GURH) between December 02, 2010 and November 30, 2016 were assessed against the inclusion criteria. In line with previous studies, patients had to be 18 years or above and met the Framingham criteria for the diagnosis of heart failure (presence of either two major criteria or combination of one major criterion and two minor criteria) [18]. The New York Heart Association functional class (NYHA) assessment was also used as an inclusion criterion. Symptomatic patients with NYHA class of either III or IV were included in the study. Moreover, robust records of patient’s laboratory and diagnostic findings such as serum sodium, creatinine, hemoglobin, hematocrit, and echocardiography were considered as a requirement of inclusion. Patients who had infections or other diseases (apart from HF) were excluded. From the available 980 patients, more than a third (n = 388) met the inclusion criteria for the study.

Patients were categorized depending on their level of sodium on the first admission to the internal medicine department. Each patient was assigned to either hyponatremia, if sodium <135mmol/L, or normonatremia, if sodium ≥135mmol/L [19, 20]. Hemoglobin concentration was used as an indicator of the patient’s anemia status. As such, a hemoglobin level of less than 13 g/dl for males or less than 12 g/dl for females was considered as anemia [21].

Last hospital discharge or medication refill time was used as a vital status checkup to assess study participants survival status. Hypertension was determined by a systolic blood pressure of greater than 140 mmHg and diastolic blood pressure more than 90 mmHg. The primary endpoint was the HF patients’ prognostic sodium level status and the secondary endpoint was all-cause mortality in HF patients.

### Statistical analysis

Continuous data like age, blood pressure (systolic and diastolic), left ventricular ejection fraction, and the serum level of laboratory findings (sodium, creatinine, hemoglobin, and hematocrit) were presented with mean and standard deviation. Categorical variables such as gender, residence, the cause of heart failure, type of medication, and NYHA class were described as percentages.

The mean difference of continuous variables among hyponatremic and normonatremia patients was calculated using a parametric statistics independent t-test. A chi-square test was performed for the discrete variables among the study groups.

To assess all-cause mortality, both univariate and multivariate Cox regression was performed. Variables with a p-value of less than 0.2 in the univariate Cox regression analysis were included for further analysis in the multivariate Cox regression analysis.

A nonparametric test, Kaplan-Meier survival analysis, was also employed to assess event-free survival among study groups. Mantel log-rank test was used to test for statistical significance.

For both independent t-test and Cox regression analyses, a 95% confidence interval was assumed. For all statistical analyses, a p-value of less than 0.05 was considered statistically significant. Statistical analysis was carried out using the Statistical Package for Social Science, version 20.0 for Windows (SPSS, Chicago, IL, USA). During the study, the patients’ data was de–identified to protect the anonymity of medical records.

### Ethical approval and consent to participate

Due to the nature of the study (retrospective data collection), ethical approval for consulting the patient’s informed consent was not deemed necessary.

## Result

The proportion of study participant in each group was comparable, with 198 (51.03%) and 190 (49.97%), in hyponatremia and normonatremia, respectively. Table 1 shows the baseline clinical characteristics of the two groups. The mean age of the participants was 54.71 (± 17.82) years. There was no significant disparity in the etiology of heart failure except for valvular heart disease (VHD) (46.97% Vs. 51.58%, P = 0.025) and other etiologies (11 Vs. 23, P = 0.023).

**Table 1:** Clinical characteristics of heart failure patients based on sodium status.

Variable		Sodium < 135 mEq/L (198)	Sodium ≥ 135 mEq/L (190)	P–Value
Age, mean ± SD		53.43 ± 18.26	56.06 ± 17.30	0.148
Gender, n (%)				0.408
	Male	80 (40.40)	69 (36.32)	
	Female	118 (59.60)	121 (63.68)	
Residency, n (%)				0.624
	Urban	83 (41.92)	75 (39.47)	
	Rural	115 (58.08)	115 (60.53)	
NYHA Class, n (%)				0.732
	Class III	44 (22.22)	45 (23.68)	
	Class IV	154 (77.78)	145 (76.32)	
Hypertension, n (%)		57 (28.79)	65 (32.83)	0.250
AF, n (%)		57 (28.79)	42 (22.11)	0.131
Heart Rate, mean ± SD		94.82 ± 18.10	90.10 ± 20.38	**0.017**
Systolic BP, mean ± SD		120.59 ± 23.60	123 ± 24.07	0.221
Diastolic BP, mean ± SD		79.38 ± 15.11	78.68 ± 14.28	0.642
Etiology of HF, n (%)				
	IHD	33 (16.67)	29 (15.26)	0.706
	VHD	93 (46.97)	68 (51.58)	**0.025**
	HHD	25 (12.63)	36 (18.95)	0.087
	DCMP	27 (13.64)	22 (11.59)	0.542
	CorPulmonary	9 (0.045)	12 (0.063)	0.44
	Other etiology	11 (0.056)	23 (0.12)	**0.023**

AF: Atrial Fibrillation, BP: Blood Pressure, DCMP: Dilated Cardiomyopathy, HF: Heart Failure, HHD: Hypertensive heart Disease, IHD: Ischemic Heart Disease, NYHA: New York Heart Association, SD: Standard Deviation, VHD: Valvular Heart Disease

### Results of laboratory analysis and echocardiograms

From the patients’ medical records, it could be observed that, as expected, there had been higher serum concertation of sodium in normonatremia groups (139.00 ± 3.68 Vs. 130.17 ± 4.00). Conversely, lower serum creatinine levels were found in normonatremia patients (0.99 ± 0.74 Vs. 1.28). All other laboratory and echocardiographic results were not statistically significant as presented in Table 2.

**Table 2:** Laboratory and echocardiography results of heart failure patients based on sodium status.

Variable	Sodium < 135 mEq/L (198)	Sodium ≥ 135 mEq/L (190)	P–Value
Hemoglobin (mean ± SD)	12.47±3.04	12.81±3.02	0.281
Creatinine (mean ± SD)	1.28 ± 0.79	0.99 ± 0.74	**<0.0001**
Sodium (mean ± SD)	130.17 ± 4.00	139.00 ± 3.68	**<0.0001**
LVEF (mean ± SD)	52.22 ± 13.79	52.34 ± 14.17	0.933

LVEF: Left ventricular Ejection Fraction, SD: Standard Deviation

### Medical treatment

Regarding the medication profile of the study cohorts, diuretics, spironolactone, and digoxin were more frequently prescribed to patients with sodium levels of less than 135 mEq/L. On the contrary, angiotensin-converting enzyme inhibitors (ACEIs) were more often prescribed to the study group with sodium level ≥ 135 mEq/L with a notable difference as illustrated in Table 3.

**Table 3:** Medication profile of heart failure patients based on sodium status.

Variable	Sodium < 135 mEq/L (198)	Sodium ≥ 135 mEq/L (190)	P–Value
Diuretics n (%)	186 (93.94)	166 (87.37)	**0.026**
Spironolactone n (%)	146 (73.74)	118 (62.11)	**0.014**
ACEI n (%)	82 (41.41)	103 (54.21)	**0.012**
Beta Blocker n (%)	91 (45.94)	99 (52.11)	0.226
Digoxin n (%)	59 (29.80)	36 (18.95)	**0.013**
CCB n (%)	19 (9.60)	14 (7.37)	0.432
Antiplatelet n (%)	37 (18.69)	46 (24.21)	0.185
Anticoagulants n (%)	47 (23.74)	39 (20.53)	0.447
Statin n (%)	28 (14.14)	33 (17.37)	0.383

ACEI: Angiotensin Converting Enzyme Inhibitor, CCB: Calcium Channel Blocker

### Survival analysis

**Fig 1:**
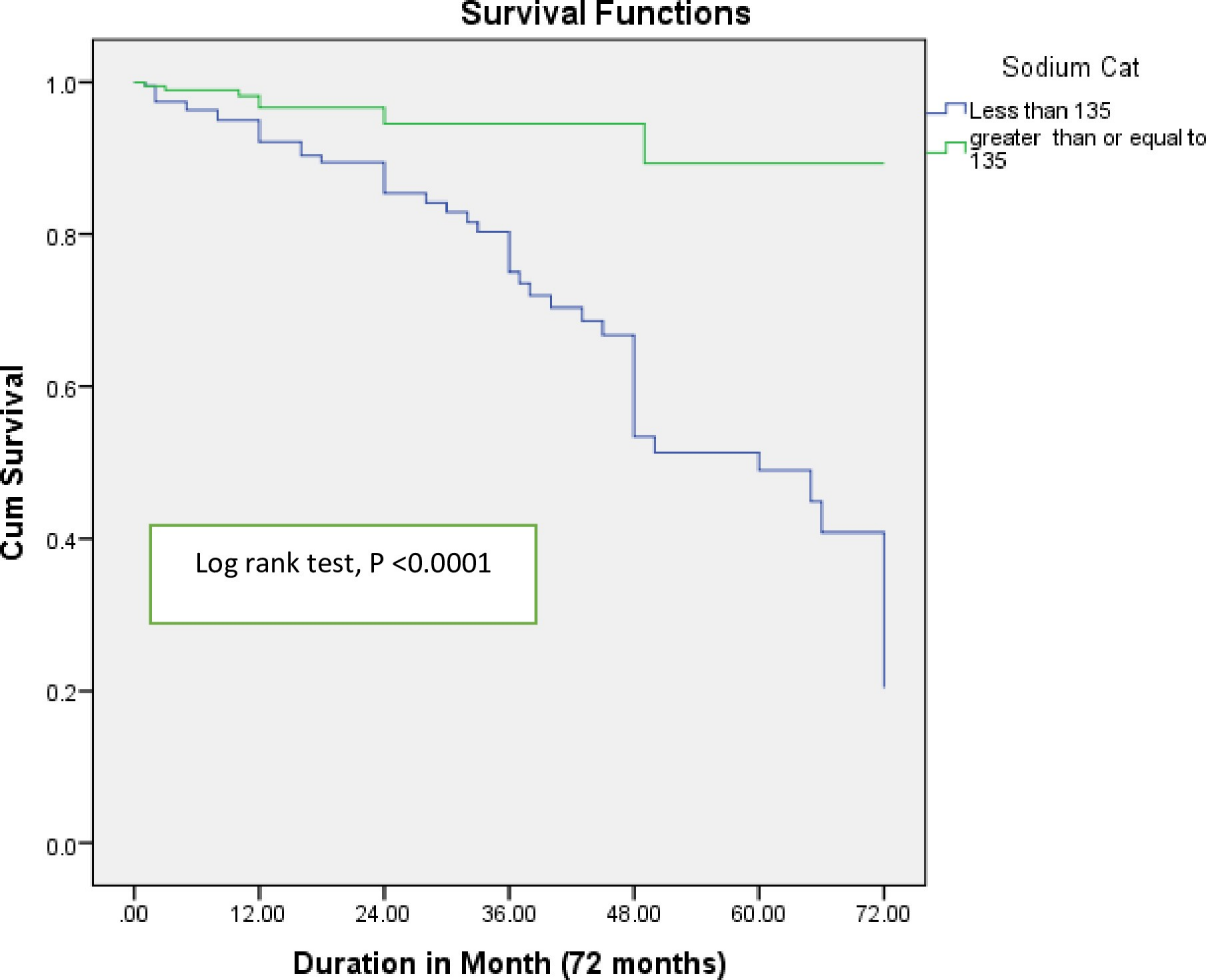
Kaplan Meier survival curves for heart failure patients based on serum sodium status. Cum Survival: Cumulative Survival; Sodium cat: Sodium category; Hyponatremia: < 135 mmol/L; Normonatremia: ≥ 135 mmol/L.

The median duration of the follow up of the study participants was 18 months (IQR = 8–36 months). The number of mortalities in the hyponatremia group was 49 patients (24.75%) compared to 8 patients observed in the normonatremia group (4.21%). Kaplan-Meier survival curves (Fig 1) showed that there was a significant difference in the survival status of HF patients among the two cohorts (Log-Rank test, P = <0.0001), patients with sodium level > 135 mEq/l having better survival rates than hyponatremic patients.

Pertinent variables with p-values less than or equal to 0.2 in the univariate Cox regression analysis were further analyzed in the multivariate Cox regression analysis as shown in Table 4. This further analysis revealed statistically significant association of mortality with the following variables: advanced age, hyponatremia, higher creatinine level and, prescription of ACEI and spironolactone. As age advances, a slight increase of mortality was observed in the study cohorts (HR 1.031 (1.008–1.055), 0.009). A similar trend was also observed with the serum creatinine; an increase of serum creatinine level has almost doubled the risk of mortality (HR 1.998 (1.556–2.566), <0.0001). Conversely, a reduction of serum sodium concentration quadrupled the risk of mortality (4.003 (1.778–9.009), 0.001). A protective effect was observed from prescription of medication. For instance, prescription of spironolactone and ACEI has significantly reduced the risk of mortality by 0.500 (0.270–0.926) and 0.412 (0.198–0.857), respectively.

**Table 4:** Predictors of all-cause mortality.

Variables		Univariate Analysis	Multivariate Analysis	
		Hazard Ratio (95% CI)	Hazard Ratio (95% CI)	P- Value
Gender	Female	1.069 (0.624–1.832)	-	
Age, Years		1.036 (1.019–1.054)	1.031 (1.008–1.055)	**0.009**
NYHA Class	Class IV	1.657 (0.783–3.506)	1.004 (0.435–2.321)	0.992
Heart Rate, bpm		1.004 (0.992–1.017)	-	
Systolic BP, mmHg		1.017 (1.008–1.026)	0.992 (0.975–1.009)	0.329
Diastolic BP, mmHg		1.031 (1.016–1.046)	1.017 (0.993–1.042)	0.169
Anemia		1.822 (1.058–3.137)	0.924 (0.487–1.753)	0.809
Hyponatremia (<135 mEq/L)	Yes	5.619 (2.659–11.872)	4.003 (1.778–9.009)	**0.001**
Creatinine, mg/dl		1.896 (1.654–2.173)	1.998 (1.556–2.566)	**<0.0001**
AF	Yes	0.568 (0.299–1.077)	2.034 (0.690–5.992)	0.198
VHD	Yes	0.695 (0.406–1.188)	0.890 (0.464–1.707)	0.727
IHD	Yes	2.179 (1.188–3.996)	0.518 (0.214–1.252)	0.144
HHD	Yes	0.887 (0.435–1.809)	-	
DCMP	Yes	0.821 (0.324–2.078)	-	
Cor pulmonare	Yes	0.731 (0.100–5.351)	-	
Other etiology	Yes	1.213 (0.435–3.379)	-	
LVEF, %		0.980 (0.960–1.001)	0.987 (0.960–1.015)	0.362
Diuretics	Yes	0.596 (0.234–1.517)	-	
Spironolactone	Yes	0.380 (0.225–0.643)	0.500 (0.270–0.926)	**0.027**
ACEI	Yes	0.525 (0.300–0.919)	0.412 (0.198–0.857)	**0.018**
Beta Blocker	Yes	0.546 (0.329–0.967)	0.670 (0.350–1.284)	0.228
Digoxin	Yes	0.431 (0.222–0.837)	0.497 (0.186–1.325)	0.162
Antiplatelates	Yes	1.147 (0.615–2.139)	-	
Anticoagulants	Yes	0.450 (0.212–0.953)	1.087 (0.389–3.038)	0.873
Statin	Yes	1.396 (0.738–2.642)	-	
CCB	Yes	1.470 (0.720–3.002)	-	

ACEI: Angiotensin Converting Enzyme Inhibitor, AF: Atrial Fibrillation, BP: Blood pressure, CCB: Calcium Channel Blocker, DCMP: Dilated Cardiomyopathy, HHD: Hypertension Heart Disease, IHD: Ischemic Heart Disease, NYHA: New York Heart Association, VHD: Valvular Heart Disease

## Discussion

Hyponatremia is a typical finding in patients with HF. Past studies of hospital-admitted patients with HF have shown a wide range of prevalence of hyponatremia, ranging from 10 to30% depending on the cut-off value used to define the condition [22–24]. In the present study, hyponatremia (< 135 mEq/L) was documented in more than half of the participants (51.03%), which is more than what was reported in most of the previous studies. The Spanish MUSIC–study, which took < 138 mmol/L as a cut-off point for hyponatremia, found a 38% prevalence of the disorder in the cohort of HF patients [25]. The difference in the prevalence between the present study and the Spanish study might be ascribed to the difference in cut-off values for hyponatremia and the inclusion of more advanced HF patients in our study.

In the current study, a significant difference was seen in the medication profile and laboratory results among hyponatremia and normonatremia patients, in particular, the use of diuretics and spironolactone. A higher level of serum creatinine was exhibited in patients with hyponatremia. Similar findings were reported by Dai-Yin Lu et al and J.C Arevalo Lorido et al [26, 27], where most of the patients with hyponatremia were found to be treated with diuretics and spironolactone and had elevated level of creatinine.

Valvular heart disease was an important etiology which was more pronounced in patients with normonatremia. The etiological significance of VHD for the development of heart failure is further supported by the Valirie N. Agbor et al. study (a meta-analysis and systematic review of heart failure etiologies in sub-Saharan Africa) where VHD (14.1%) was the third leading cause of HF preceded by hypertensive heart disease (39.2%) and cardiomyopathy (21.4%) [28]. Tefera et al. further showed the valvular implication of VHD with findings of mitral regurgitation and mitral stenosis as the most common valvular involvement in Ethiopian HF cohorts [29]. In contrast, the incidence of VHD was 63.9 per 100,000 person-years in a Swedish nationwide hospital-based registry study, with aortic stenosis (47.2%), mitral regurgitation (24.2%) and aortic regurgitation (18%) being the most common valvular involvements [30].

There have been varying outcomes in the prognostic impact of hyponatremia in several studies. In the Heart Failure Registry of Taipei Veterans General Hospital (HARVEST) study, serum sodium was found to be an independent risk factor for all-cause mortality in outpatients with HF [26]. Likewise, the Duke Databank for Cardiovascular Diseases (DDCD) study reported that plasma sodium <135 mmol/L was associated with overall mortality, cardiovascular mortality or rehospitalization [23]. Conversely, the pioneering EVEREST study pointed out that attempts at correcting low serum sodium levels did not improve survival. However, it should be emphasized that in the EVEREST studies, tolvaptan, a vasopressin receptor antagonist, increased serum sodium by enhancing free water clearance at the level of kidneys. Further, the study included patients with NYHA class III-IV HF under both scenarios (i.e., in the event that they had hyponatremia or normal serum sodium levels at admission) and therefore, in the EVEREST studies, hyponatremia was not a requirement for admission into study.

In this pool of patients with variable serum sodium levels, tolvaptan did not demonstrate an improvement in survival due to an increase in sodium level or a reduction in dyspnea [27, 31]. Nonetheless, there are additional studies in which heart failure patients, all hyponatremic at the time of hospital admission, were evaluated with regard to short-term mortality. A significant decrease in mortality was reported in the group that had their serum sodium normalized. In the current study, even though the levels of sodium on admission was not exclusively < 135 mmol/L, hyponatremia did predict mortality in HF patients [23, 26]. Furthermore, Kaplan-Meier survival curve showed (Log-Rank test, P < 0.0001) a worse survival prognosis in the hyponatremia group of HF patients, which is in alignment with the above studies [24, 26, 27].

The pathogenesis of hyponatremia in HF is considered to be multidimensional and correlated to disease severity [32]. In most HF patients’ cases, hypervolemic-hyponatremia is the common denominator or nexus. The interlinking of increased secretion of arginine vasopressin (AVP)-enhanced activity of the sympathetic nervous system and the renin-angiotensin system plays a paramount factor in the development of hyponatremia [33, 34].

Another point of interest is the significant difference in the prescription of digoxin, as shown in Table 3; hyponatremic patients tend to take this medication more frequently than normonatremic patients. This might be attributed to a relatively higher proportion of atrial fibrillation (AF) in the hyponatremic cohorts, though it was not significant. As supported by numerous studies, digoxin is commonly prescribed for patients with heart failure and AF [35, 36] but circumspection is necessary for the potential adverse drug reactions in compromised renal function–cardiorenal patients and frequent creatinine clearance measurement is mandatory in tailoring the dose in accordance to the glomerular filtration rate. As Shlipak MG et al. demonstrated in the digitalis intervention trial, there is an increased annual mortality when eGFR is reduced to below 50 ml/ml/1.73m^2^ [37].

In our study, independent prognostic markers of all-cause mortality among study participants were hyponatremia (AHR = 4.003 (1.778–9.009), P = 0.001), advanced age (AHR = 1.035 (1.012–1.058), P = 0.003), higher creatinine level (AHR = 1.929 (1.523–2.443), P = <0.0001), and prescription of medications like, ACEI (AHR = 0.410 (0.199–0.842), P = 0.015) and spironolactone (AHR = 0.511 (0.275–0.949), P = 0.033). These findings were on par with studies conducted in Ethiopia [16], Poland [38], Spain [39, 40], the UK [41], and the US [42] that showed an unfavorable prognosis in HF cohorts, who were at advanced ages and had lower levels of sodium and higher serum creatinine levels. A randomized controlled trial on the effect of a known aldosterone blocker, spironolactone, on HF patients’ survival revealed the paramount impact on morbidity and mortality by lowering the atrial natriuretic peptide concentrations [43]. In a recent European cohort study, prescription of ACEI in HF treatment significantly decreased mortality in patients with HF [38].

Different strategies are in place depending on the characteristics of the patient cohort to manage hyponatremia in HF patients. Although these strategies are not the scope of the current study, a brief discussion of modalities may provide vital information on managing hyponatremia. In HF patients with acute symptomatic hyponatremia and neurologic symptoms due to brain edema (resulting from fluid shifts from the hypotonic extracellular fluid into the more hypertonic brain), immediate treatment is paramount to prevent or reduce the risk of neurologic complications [44–46]. The typical management for this scenario is to administer an infusion of hypertonic saline, setting the dose at 1–2 mEq/L per hour until neurological symptoms improve [47]. After the symptoms subside, chronic management of hyponatremia should be implemented. However, hyponatremia should be gradually corrected with a serum sodium correction of less than or equal to 8 mEq/L per 24 hour [48, 49] as a more aggressive correction may expose patients to central pontine myelinoylsis [44, 50].

In patients with chronic hyponatremia with no neurological symptoms, there are different treatment options. The first and least expensive option is a fluid restriction amounting to less than 800–1000 mL/day to achieve a negative water balance [47]. In a randomized control trial, hyponatremic patients (≤ 137 mg/dl) with a daily fluid of < 1000 ml had better symptom reduction and overall quality of life [51]. Conversely, compliance with fluid restriction is the main obstacle to the effectiveness of this treatment option [47].

The second option is diuretics treatment; loop diuretics are the mainstay medication in HF with fluid overload [52]. Addition of furosemide with an ACEI has significantly improved sodium concertation [53, 54]. Moreover, co-administering hypertonic saline infusion with a high dose of diuretics has shown a potentially better outcome and increased sodium level in HF patients [55, 56]. In a study conducted by Paterna S et al. in a cohort where patients with NYHA class IV received an infusion of furosemide (500–1000 mg) and hypertonic saline (150 ml 1.4% - 4.6% NaCl) every 30 min for 6 to 12 days [55], an increase in sodium levels, reduced hospital stays, and decreased readmission rates were seen in comparison to furosemide infusion alone [55]. This finding is further supported by a large sample size study of similar patient characteristics [56].

The third alternative, the recent treatment modality with large clinical interest, is the arginine vasopressin (AVP) receptor antagonists [57], which has three receptors (V1A, V1B, and V2) [57]. Among these receptors, the V2 receptors are important in the development of hyponatremia in HF patients. V2 receptors are mainly found in the renal collecting ducts and have the role of free water reabsorption resulting in enhanced water retention [58]. AVP receptor antagonists (vaptans) play the central role in regulating water retention by these receptors [59]. In the Acute and Chronic Therapeutic Impact of a Vasopressin Antagonist in Congestive Heart Failure (ACTIV in CHF) trial, tolvaptan administration in a patient with systolic dysfunction resulted in lowering of body weight in 24 hours without impacting the blood pressure, heart rate or furthering the rate of hypokalemia or deteriorating renal function [60].

Another study on tolvaptan, the EVEREST investigation, showed a significant weight reduction on day 7 of hospital discharge in patients treated with tolvaptan [11]. Even though tolvaptan showed a reduction of body weight, normalization of sodium level and relief of dyspnea, some studies indicated that administration of tolvaptan did not show long-term mortality reduction or heart failure-related morbidity [61]. More clinical studies are needed to further address the long-term impact of AVP receptor antagonists in selected patients (hyponatremic and/or elevated AVP level) with HF.

Various limitations have been presented in our study. An important laboratory parameter, NT-proBNP, was not recorded due to unavailability in the patients’ medical records. Another important limitation was the assumption taken for the survival analysis, that the last hospital discharge or last medication refill date was considered as the final follow up time. Telephone contact with study patients was hindered due to the absence of contact addresses in the medical records. Further, assessment of the impact of persistent hyponatremia, as well as corrected hyponatremia would have been possible if sodium levels beyond admission had been considered. In addition, admission diuretic dose was not collected and consequently, a potential association between diuretic dose and sodium level could not be examined. As other prognostic factors (i.e. medication, creatine level, and age) also played vital roles in overall survival, well-controlled clinical trials (complete with medication dosing, laboratory outputs and long-term prospective follow up) are required to further study the impact of hyponatremia in HF patient’s prognosis in low income nations. Lastly, the paucity of multicenter data and small sample size made it difficult to generalize the study findings to represent the current scenario nationwide.

In conclusion, despite these limitations, we believe that our study provides important insights into the clinical features and prognosis of HF patients with hyponatremia in Ethiopia. The disease burden of HF has a significant impact on patients’ morbidity; moreover, the economical aspect of treating HF is also an important aspect, which necessitate further study on the financial burden the disease imposes on HF patients.
