# Correction: The prognosis of heart failure patients: Does sodium level play a significant role?

**DOI:** 10.1371/journal.pone.0223007

**Published:** 2019-09-19

**Authors:** Tamrat Befekadu Abebe, Eyob Alemayehu Gebreyohannes, Yonas Getaye Tefera, Akshaya Srikanth Bhagavathula, Daniel Asfaw Erku, Sewunet Admasu Belachew, Begashaw Melaku Gebresillassie, Tadesse Melaku Abegaz

The supplementary dataset is omitted from the list of Supporting Information. It can be viewed below as S1 File.

## Supporting information

S1 File Supplementary dataset. (CSV)
